# 

**Published:** 1895-11

**Authors:** 


### CONTENTS.

### COMMUNICATIONS.

Rhisodontrypy, or Hullihen's Operation.... 521
Abrasion of the Teeth.... 532
President's Address.... 535
Ought the Formation of Dental Schools be Limited?.... 536
Business Education for Professional Men.... 537
The Aesthetic Correction of Facial Contours in the Practice of Dental Orthopedia.... 540
Dentistry in Brazil.... 562

### REPORT.

Report of Treasurer of World's Columbian Dental Congress in full from March 23d, 1891, to July 23d, 1895.... 564

### SELECTIONS.

The Health of a Bacillus.... 565
The Teachings of Adversity.... 561
An Illegal Practitioner sent to Jail.... 563
Phosphorus Poisoning.... 566
An Avalanche of Modern Drugs.... 567
Treatment of Phlegmonous Inflammations.... 569

### EDITORIAL.

Ohio State Dental Society.... 569
The American Dentist in Europe.... 570
Ohio State Dental Society.... 571
Bibliographical.... 572

